# Family and Individual Quality of Life in Parents of Children with Developmental Disorders and Diabetes Type 1

**DOI:** 10.3390/jcm11102861

**Published:** 2022-05-19

**Authors:** Marija Ljubičić, Sanja Delin, Ivana Kolčić

**Affiliations:** 1 Department of Health Studies, University of Zadar, Splitska 1, 23000 Zadar, Croatia; 2 Department of Pediatrics, General Hospital Zadar, Bože Peričića 5, 23000 Zadar, Croatia; delinsanja@gmail.com; 3 Department of Psychology, University of Zadar, Obala kralja Petra Krešimira IV 2, 23000 Zadar, Croatia; 4 Department of Public Health, University of Split School of Medicine, 21000 Split, Croatia; ikolcic@mefst.hr or; 5 Algebra LAB, Algebra University College, Gradišćanska ul. 24, 10000 Zagreb, Croatia

**Keywords:** quality of life, parents, children with disabilities, children with diabetes type 1, mental health

## Abstract

Background: This cross-sectional study assessed both family and individual quality of life (QOL), and their association with self-esteem, optimism, chronic psychological stress, anxiety, and depression in parents of children with chronic conditions. Methods: Parents of children with Down syndrome (DS), autistic spectrum disorder (ASD), cerebral palsy (CP), diabetes mellitus type 1 (DMT1), and parents of children without chronic diseases with typical development (TD) were included. Multivariate linear regression analysis was used to assess parental characteristics associated with the domains of individual and family QOL. Results: Compared to the parents of TD children, parents of children with ASD and DS were more likely to report reduced family QOL in all domains, while parents of children with DMT1 had lower parental perception. Self-esteem was positively associated with all domains of individual QOL, while optimism was associated with the overall individual QOL perception and health. Higher stress perception was negatively associated with most of the domains of individual and family QOL. Conclusions: This study confirmed that parents of children with chronic conditions are more likely to have lower perception of both individual and family QOL, which were associated with self-esteem, chronic stress, anxiety, and depression. Interventions should focus not only on the child with a chronic condition but on parents too.

## 1. Introduction

Parenting is a unique life experience, which ideally includes raising a child in an atmosphere of love and in a supportive and stimulating family environment. However, the birth of a child with a disability or onset of a chronic illness in a previously healthy child can hinder the family balance, disrupt a positive atmosphere, the usual family routine, and the quality of life (QOL) [1]. According to the World Health Organization, individual quality of life is defined as “individuals perceptions of their position in life in the context of culture and value systems in which they live and in relation to their expectations, goals and standards” [2]. On the other hand, the family quality of life represents the individual experience of own quality of life within the family context, as well as possibilities of the whole family to achieve its goals in the society [3].

The family home becomes the place of constant care for a child with a chronic condition [4]. Taking care of such a child can be challenging, because the parents need to acquire knowledge and skills in performing specific and sometimes highly complex interventions. These parents can never take a real break, and they sometimes get the feeling that taking care of their child takes up more time and energy than they can really give [4]. This may prompt them to re-examine whether they are sufficiently effective in their parental role. Additionally, the inability to balance between different obligations can result in feelings of guilt and mourning of the lost opportunities, demotivation for daily activities, low satisfaction and feeling of vitality, a negative self-perception and low self-esteem, as well as depression and reduced quality of life [5,6,7,8]. Even though most families adapt to the situation over time and find their own routine, it has been confirmed that parental perception of the quality of life depends on the severity of the illness and the extent of disability of the child [9,10].

Developmental difficulties such as Down syndrome (DS), autistic spectrum disorders (ASD) and cerebral palsy (CP) are among the most common chronic conditions in childhood [11]. It was shown that parents of children with ASD have higher risk for experiencing chronic psychological stress, and thus impaired QOL, in comparison to parents of children with typical development (TD) and without chronic diseases [7,12,13], and compared to parents of children with Down syndrome [14]. A systematic review showed that parents of children with infantile cerebral palsy had lower QOL, along with higher levels of stress and depression, compared to the parents of TD children [10]. Additionally, parents of children with diabetes type 1 (DMT1) often worry about the possibility of the hypoglycemic episodes in their children, whereas the stronger the stress perception, the greater the negative influence on the parent’s health and the QOL [15]. The health-related needs in children with disabilities and diabetes are very complex and possibly challenging for parents. For example, the complex tasks in parents of children with disabilities related to the developmental habilitation, such as early intervention, physical therapy, speech therapy, and sensory integration are as obligatory as the implementation of self-control, nutrition and physical activity in children with diabetes. On the other hand, hypoglycemia can be the reason for continuous parental fear, tension and worry, not only for the child’s future but also for the child’s life. Excessive responsibilities in the parents of children with diabetes can disrupt family harmony just as much as the complex setbacks related to a child’s disability. However, to the best of our knowledge, no study has so far compared the QOL in parents of children with developmental disabilities with QOL in parents of children with DMT1, while comparing both parental groups to parents of TD children. Additionally, only a small number of studies have assessed both the individual and the family QOL in parents of children with chronic conditions [16]. Furthermore, previous studies indicate a lack of comprehensive understanding of the QOL in parents of children with chronic conditions, while such understanding is required in order to identify the parents in need of timely support [17]. Most studies on parenting a child with disability identified the child’s characteristics, impairment and related parental obligations as stressors [18]. Nevertheless, whilst this stress is undeniable, several studies reported that carers can experience both stress and positive experiences simultaneously [18]. For example, a study in parents of children with DS found that these parents are happy, love their child with DS, and that their children are great source of love and pride [19]. Furthermore, most of the QOL studies examined the physical and psychological health while considering the social relationships, but not addressing the specifics about family functioning. Lastly, studies on the association between self-esteem, optimism, anxiety and depression, and the family QOL in parents of children with chronic conditions are scarce in the literature. Self-esteem has been reported to have a mediating role for the QOL perception, and studies confirmed association between optimism, depression, anxiety and self-esteem [20,21,22,23]. However, these characteristics in parents of children with DS, ASD, CP, and DMT1, and their association with family QOL are not represented in the literature. Due to this gap in knowledge, further studies are needed in order to identify the factors that determine parental QOL in families with a child with chronic conditions [10]. The lack of evidence on quality-of-life outcomes among parents of children with disability and DMT1 warrants future studies.

The aim of this study was to assess the individual and the family QOL in parents of children with ASD, DS, CP, and DMT1 in comparison to parents of TD children without chronic diseases, while taking into consideration self-esteem, optimism, stress, anxiety, and depression. We hypothesize lower parental individual QOL and family QOL in parents of children with chronic conditions.

## 2. Materials and Methods

### 2.1. Sample and Settings

This cross-sectional study was conducted between March 2018 and March 2019, and it included parents of children aged 4 to 12 years (*N* = 244), while children were not included. Detailed protocol of the study was previously described [24]. In short, the protocol of the study was explained to all potential examinees before inclusion in the study, as well as the risks and benefits of participation, after which nine parents withdrew. The final sample included 235 examinees (91 fathers and 144 mothers; response rate 96.3%). The examinees were allocated to five groups: parents of children with ASD, DS, CP, and DMT1 (51 fathers and 91 mothers in total), and a CG of parents of TD children without chronic conditions (40 fathers and 53 mothers).

These disability groups and DMT1 group were selected in order to represent diverse health-related challenges for both children and parents, and based on their either common (ASD and DS) or less common (DMT1 and CP) representation in the literature.

The diagnoses of the children were based on standard healthcare procedures, according to the ICD-10 (F84.0 and F84.1 for ASD, Q90 for DS, G80 for CP, and E10 for DMT1). All the diagnoses were confirmed by specialists.

Parents of children with DS, ASD, CP and DMT1 were recruited from Zadar General Hospital, pediatric departments, kindergartens and patients’ associations. Parents of the TD children were recruited from the same target population, namely the same kindergartens, schools and pediatric departments, to ensure their comparability to other study groups.

Inclusion criteria were parents with one child with DDs or DMT1, and that child could have only one diagnosis obtained at least 6 months prior to the study enrolment. Exclusion criteria were parents with more than one child with DDs and/or DMT1, presence of two or more chronic conditions (i.e., if the same child had a diagnosis of DS and ASD), presence of other disabilities or chronic diseases in a child, and the child’s diagnosis reached in less than 6 months prior to the parental enrolment in the study. Parents with psychological and severe chronic conditions were excluded, as well as those with malignant diseases, those taking sedatives, anxiolytics, and corticosteroids, working night shifts, pregnant and lactating women (actively or completed within less than 6 months), parents whose child’s diagnosis was made within the last 6 months, parents of children with multiple difficulties and more chronic and/or rare diseases. After considering these factors, 11 examinees were excluded. Additionally, seven parents refused to fill out the questionnaire without giving an explanation (four parents of children with TD and without chronic diseases; two parents of children with ASD; and one parent of a child with DMT1).

This study was performed in accordance with the Declaration of Helsinki ethical standards. All participants signed a consent form, received oral and written instructions on the course of the study and the protection of privacy. The study was approved by the Ethical Committee of the Zadar General Hospital (01-3942/18-7/18) and the Ethical Committee of the University of Split, School of Medicine (registration number 2181-198-03-04-18-0014). The study was registered in the ClinicalTrials.gov (ID: NCT03602378).

### 2.2. Data Collection

We collected information about gender, age, number of children in the family and their age, marital status, education, work status, income, associations’ membership, medical history and lifestyle habits. We used a questionnaire to assess the family QOL, individual QOL, stress perception, depression, anxiety, optimism and self-esteem of the parents.

#### 2.2.1. Medical History and Lifestyle Habits

Medical history included previous diagnoses of chronic diseases (coronary heart disease, diabetes, hypertension, hyperlipidemia, obstructive pulmonary disease, gastrointestinal, kidney, thyroid, autoimmune and musculoskeletal diseases, and malignancy). We summed all of these possible chronic conditions in each parent to define a burden of chronic diseases (none, one, two or more diseases). We measured body mass and height using a mechanical seca 700 scale (seca GmbH & co. kg, Hamburg, Germany; with a precision of 50 g and a capacity of 220 kg for body mass, and a measuring rod with a range between 60 and 220 cm for body height). Body mass index (BMI) was calculated (kg/m^2^).

Lifestyle assessment included smoking, alcohol consumption, Mediterranean diet and sitting activity. Mediterranean Dietary Serving Score (MDSS) included 14 food groups (cereals, vegetables, fruit, olive oil, nuts, milk, red meat, white meat, eggs, fish, legumes, potatoes, red wine, and sweets), and this short questionnaire was validated for use in adults from Croatia [25]. MDSS score estimates the compliance with the Mediterranean diet (MD) with the maximum score of 24 points, while cut-off score was ≥14 points [26].

The short form of the International Physical Activity Questionnaires (IPAQ) was used to estimate average daily sitting time during the last seven days [27].

#### 2.2.2. Questionnaires

The Beach Center Family Quality of Life Scale (FQOL) assesses the perception of family quality of life in families with children with disabilities [28]. The scale contains 25 questions divided into five subscales: family interaction, parenting, emotional well-being, physical/material well-being, and disability-related support. The scoring for each question ranges between 1 (very dissatisfied) and 5 (very satisfied). The overall family QOL was divided into 3 categories: low (25–58 points), medium (59–91 points), and high level (92–125) [28]. Cronbach’s alpha for this questionnaire applied in our sample was α = 0.952.

The World Health Organization Quality of Life scale (WHOQOL-BREF) is a frequently used instrument for assessment of the QOL, which is comprised of 26 items ranked on a 5-point Likert type scale [2]. The 24 items are quantifying four domains (physical health, psychological well-being, social relationships, and environmental support). Two items assess the general QOL and satisfaction with own health, and these are not included in the domain scoring. The possible answers for each question ranged between 1 (very dissatisfied/very poor) and 5 (very satisfied/very good). Potential scores ranged between 4 and 20, with higher score denoting better QOL [2]. Cronbach’s alpha achieved in the entire sample within this study was α = 0.922.

Rosenberg Self-Esteem Scale (RSES) is used for assessing general feelings about oneself. It consist of 10 items scored on 4-point Likert type scale (3—strongly agree, 2—agree, 1—disagree, 0—strongly disagree), with the overall possible scores in the range 0–30, where higher score corresponds to greater level of self-esteem [29]. Cronbach’s alpha was α = 0.856.

Life Orientation Test–Revised (LOT-R) is a 10-item instrument for assessing dispositional optimism. Of the 10 items, 3 items assess optimism, 3 items assess pessimism, and 4 items serve as fillers, using a Likert scale ranging from 0 (agree a lot) to 4 (disagree a lot). Scoring is kept continuous [30]. Cronbach’s alpha was α = 0.752.

Patient Health Questionnaire scale (PHQ-9) is a 9-item scale intended to probe the frequency of depressive experiences over the last 2 weeks. Items are ranked on a 4-point Likert scale (0—not at all; 1—several days, 2—more than half the days, 3—nearly every day). Scores of 0–4 represent none to minimal depression, 5–9 mild, 10–14 moderate depression, 15–19 moderately severe, while 20–27 represents severe depression [31]. Cronbach’s alpha was α = 0.855.

Generalized Anxiety Disorder Scale (GAD-7) consists of 7 items assessing worry and anxiety symptoms as nervous, anxious, trouble of relaxing and irritable over the last 2 weeks. Items are scored on a 4-point scale (0—not at all; 1—several days, 2—more than half the days, 3—nearly every day). Scores of 0–4 represent none to minimal anxiety; 5–9 mild; 10–14 moderate; 15–21 severe anxiety [32]. Cronbach’s alpha was α = 0.896.

Perceived Stress Scale (PSS-10) is an instrument with 10 items used for assessing the perception of stress during the past month. Each item was ranked using the 5-point Likert type scale (0—never, 1—almost never, 2—sometimes, 3—fairly often, 4—very often). The overall possible score ranges from 0–40, and higher scores predict greater level of perceived stress [33]. Cronbach’s alpha in our sample was α = 0.879.

#### 2.2.3. Statistical Analysis

The analysis was performed using a final sample of 217 parents. We used the Shapiro–Wilk test to assess data distribution. Depending on the distribution, we calculated the mean and standard deviation or median and interquartile range for the numerical variables. Absolute numbers and percentages were used for description of categorical variables. We investigated difference between groups using chi-square test, ANOVA with LSD post hoc test for normal distribution, or Kruskal–Wallis test with Mann–Whitney U test as a post hoc test when distribution was non-normal. Cronbach’s alpha was used to assess the internal consistency of the questionnaires.

The correlation between variables was analyzed using the recoded Pearson correlation coefficient (non-partial correlation was used to adjust for gender and age). We performed a Spearman’s partial correlation on non-normally distributed data, followed by conversion of Spearman rho coefficients to Pearson correlation coefficients.

Finally, we created several multivariate linear regression models to assess the association between individual and family QOL perception with child’s diagnosis, parental self-esteem, optimism, anxiety, depression, and stress perception. Dependent variables were all domains of individual and family QOL (separated in each model), while several predictor variables were included in each model as independent variables: study group (control group was a reference group), parent’s gender (mothers were reference group), parent’s education (university level was reference group), parent’s age, smoking (active smokers were reference group), chronic diseases (yes was reference group), monthly income (>1.400 EUR was reference group), association’s membership (yes was reference group), sitting activity, Mediterranean diet, BMI, time spent in care for children, self-esteem, optimism, anxiety, depression, and stress perception. All predictors were introduced into the model simultaneously. 

Sample size calculation was performed a priori based on previous study results [34], which yielded a required sample of 11 parents per group to achieve the power of 80% (using an online calculator Available online: https://www.stat.ubc.ca/-rollin/stats/ssize/n2.html (accessed on 6 January 2018)) The data analysis was conducted using the SPSS Statistic v21.0 (IBM, Armonk, NY, USA). Statistically significant values were those with *p* < 0.05.

## 3. Results

### 3.1. Sociodemographic Characteristics of Parents and Family Composition

Compared to the CG, parents of children with chronic conditions had lower educational and economic status, and one third of parents used a legal possibility of being exempt from work due to the care for a child with chronic condition (Supplemental Table S1). Boys were predominant in the ASD group, while a higher percentage of girls was recorded in the DS group. Parents of children with ASD reported the lowest number of close friends compared to other parental groups. Parental groups did not differ significantly in time spent in caring for a child with a chronic condition, but compared to the CG, they devoted slightly less time to their other children. Compared to the CG, parents of children with DMT1, ASD and DS were more frequently members of local patient or parental associations (Supplemental Table S1).

### 3.2. Psychological Characteristics of Parents

In contrast to parents of TD children, parents of children with a chronic condition showed a significant deviation in psychological characteristics (Table 1). Parents of children with ASD and DS showed lower self-esteem, while parents of children with DMT1 and DS were the least optimistic. Compared with CG, depressive symptoms were higher in parents of children with ASD, CP and DMT1. Parents of children with ASD showed a higher level of perceived stress in comparison to CG (Table 1).

### 3.3. Family and Individual Quality of Life in Parents of Children with Chronic Conditions

Investigated groups differed in comparison to the CG in both family and individual quality of life (Table 1). Compared to the CG, parents of children with disabilities showed a lower level of family quality of life, while that was not the case for parents of children with DMT1. Family interaction was significantly lower in parents of children with DS (*p* = 0.001), and ASD (*p* < 0.001). Parents of children with ASD and DS showed a lower perception of parenting (*p* < 0.001, for both groups), with similar result for perceived lower disability-related support (DS vs. CG *p* = 0.007, and ASD vs. CG *p* < 0.001), emotional well-being and physical/material well-being (Table 1).

Compared to the CG, parents of children with disabilities showed a lower level of individual quality of life in all domains (Table 1). The lowest perception of physical health was recorded in parents of children with ASD and CP. Psychological well-being and social relationships were the lowest in parents of children with ASD. A similar result in the social relationships domain was observed in parents of children with DS and DMT1. All parents of children with disabilities reported a weaker environmental support compared to the CG, as well as a negative deviation in their individual perception of the QOL and their own health (Table 1).

### 3.4. Association of Parental Characteristics with Family and Individual Quality of Life

A moderate correlation between individual and family quality of life was demonstrated in all of the domains (Supplemental Table S2). The highest correlation was recorded between the WHOQOL psychological domain and the overall family QOL (r = 0.60; *p* < 0.001), and between the WHOQOL environment domain and the family QOL physical/material well-being domain (r = 0.61; *p* < 0.001) (Supplemental Table S2).

The results obtained using multivariate linear regression confirmed the association between the child’s condition and the family QOL (Table 2). Compared to the CG, parents of children with ASD and DS were more likely to have reduced family QOL in all of the domains, while parents of children with DMT1 had impaired parental perception (ß = −0.22; *p* = 0.032), a borderline insignificant result for disability support perception (ß = −0.18; *p* = 0.085), and reduced overall family QOL (ß = −0.18; *p* = 0.041). Parental education, association’s membership and time spent in caring for a child contributed significantly to the overall family QOL (Table 2). Lifestyle habits were not associated with the parental family QOL, except for the Mediterranean diet adherence, which was positively associated with family interaction. Self-esteem was a moderate positive predictor of better family interaction (ß = 0.38; *p* < 0.001), parenting (ß = 0.23; *p* = 0.013), and higher overall family QOL (ß = 0.24; *p* = 0.003). Anxiety displayed a positive association with the family QOL in all domains, while depression was associated negatively with parenting (ß = −0.26; *p* = 0.045), physical and material well-being (ß = −0.31; *p* = 0.012), and the overall family QOL (ß = −0.23; *p* = 0.042). Stress perception was a significant negative predictor of the family QOL in all of the domains, except in the parenting domain (Table 2).

Multivariate linear regression also confirmed the association between the individual QOL and parental groups, sociodemographic variables, lifestyle habits, and psychological characteristics of parents (Table 3). Parents of children with ASD were more likely to have disturbed social relationships (ß = −0.19; *p* = 0.026). Lower perception of the environmental support domain was recorded in parents of children with CP (ß = −0.16; *p* = 0.010). Additionally, lower individual perception of the QOL and one’s health was associated with having a child with ASD (ß = −0.16; *p* = 0.024) and CP (ß = −0.13; *p* = 0.040). Self-esteem was a moderate predictor of all individual QOL domains, especially for the domain of the psychological well-being (ß = 0.59; *p* < 0.001) and social relationships (ß = 0.42; *p* < 0.001), while optimism was associated with the overall perception of the QOL (ß = 0.25; *p* = 0.001). Although anxiety and depression were not associated with the individual QOL, perceived stress was negatively associated with all domains of the individual QOL, except for the domain of social relationships (Table 3).

## 4. Discussion

The aim of this study was to examine the family and individual QOL, and to determine their association with psychological stress, anxiety, and depression, while considering the sociodemographic and lifestyle characteristics of parents of children with disabilities and DMT1. Our results show that parents of children with ASD, DS, and DMT1 had reduced family QOL compared to parents of TD children. Additionally, parents of children with ASD had lower perception of the social relationships domain of the individual QOL and the overall individual QOL, while parents of children with CP had higher probability of lower perception of environmental support and lower overall perception of the individual QOL. Self-esteem was positively associated with all domains of the individual quality of life, while stress perception was a significant negative predictor for most domains of both individual and family QOL. Interestingly, anxiety displayed a positive association with domains of the family quality of life, while depression was associated negatively with parenting, physical and material well-being, and the overall family QOL.

The most important support for children with disabilities and those with chronic illness are their parents [35]. However, these parents endure certain sacrifices and experience various difficulties while caring for and raising their children. This could lead to a lower QOL due to reduced general well-being, demotivation, and loss of life satisfaction [35]. Studies confirm that a higher burden of caring for a child with a chronic condition is associated with higher risks of having poorer QOL [35], and this was confirmed by our results in the domains of family QOL, as well as in individual QOL.

Lower family interaction, weaker perception of parental roles within the family structure, and greater deviations in the emotional, physical and material well-being in parents with ASD and DS can be explained by depression and lower self-esteem. Such outcomes were not observed in parents of children with DMT1, although their care for children is also never-ending and required throughout the day. On the other hand, their optimism was impaired more than in other study groups. It is possible that fear of hypoglycemia and the need to adapt on a daily basis have a negative impact on parental optimism. Although some studies found difficult verbalization of feelings within families with children diagnosed with DMT1 [36], our results may indicate mutual support, care, and participation of all family members in achieving better control of the child’s illness and making important disease-related decisions. However, the parenthood was impaired in groups of chronically ill children, except in the CP group. It is possible that due to the child’s condition they do not find enough time to take care of the needs of each individual child, which was confirmed by the contribution of the time spent in childcare to all the domains of the family QOL. For example, DMT1 permeates all life activities [37], and it is often characterized as a “family disease” that significantly affects the way the family functions and interacts [38]. Such previous findings may explain our results pointing to a significant contribution of the care for a child with DMT1 to the parenting domain and the overall family QOL. Additionally, the lack of disability support in parents with ASD and DS may induce higher parental stress, which can be supported by our finding of association between disability support and perceived stress.

Additionally, it is possible that a lack of support from friends and feeling of rejection by others increase social isolation and disrupted well-being [39]. Such an interpretation is also confirmed by the fact that parents of children with ASD reported to have the lowest number of friends in whom they can confide. This possibly results in weaker social relationships of parents of children with ASD, which indicates that both family-related and social factors are important for parenting. Moreover, stressors that favor impaired family QOL include social isolation, low partner support, low social support satisfaction, and inadequate support from system services [40].

Families’ material well-being segment, which includes health support and a sense of security for all the family members in all environments in which they reside, was also impaired in parents of children with DS and ASD, significantly more so than in the CG. Association of this domain with anxiety, depression and perceived stress may point to the lack of social and health support. It is possible that parents of children with disabilities, especially those with ASD, often encounter problems in the health system and are faced with many challenges while receiving medical care [41]. The studies indicate that families are pivotal in early intervention and child development [42]. However, the expert advice they receive may not be enough for them to have a complete sense of security in childcare, and they often feel left to fend for themselves and without skills of parental advocacy [43]. Additionally, the guidelines they receive often have to be modified according to the child’s condition and unpredictable behavior, and very often they perceive circumstances beyond their own control, emphasizing numerous difficulties and unmet psychological and clinical needs of their children and themselves personally. Although in our study the parenting of a child with CP was not associated with the family’s material well-being, studies confirm that parents of children with CP, due to the limited mobility of the child and the use of mobility aids, often have difficulties compared to parents of TD children, but also to those with ASD, DS and DMT1 [44]. Indeed, this was recorded in our study in the domain of the environment within the individual QOL.

Lower parental perception of the disability-related support domain within family QOL may indicate that the families of these children do not have sufficient support in achieving their goals at school and in the workplace, and that support systems in society are not clearly emphasized to facilitate the care for these children [34]. Additionally, the parents of these children, especially those with DMT1, must acquire the knowledge and skills that are within the competence of health professionals, such as blood glucose checks, multiple daily administrations of insulin injections, recalculation of caloric intake and required insulin units, ways of preventing hypoglycemia, and control of physical activity [15]. Such activities require a high level of responsibility and skills, which can be the source of higher stress. 

Furthermore, the family is the most immediate and the most important environment for the child’s growth and socialization, and family togetherness and the way the family functions are considered to be important factors in the development of self-esteem [45]. Our results confirm a strong association between family interaction and self-esteem, which indicates that family relationships are extremely important for parental self-esteem as well, and vice versa. Studies confirm the association between self-esteem and family relationships, where low self-esteem and poorer mental health were associated with depression [45]. Our study confirmed the importance of self-esteem in almost all the areas of both individual and family QOL. This confirms the results of previous studies showing that self-esteem is one of the strongest predictors of subjective well-being, life satisfaction and QOL [46,47]. Self-esteem is extremely important in fulfilling the parental role, primarily because higher self-esteem in the parent–child interaction can improve the child’s subjective well-being [48]. In our study, parents of children with ASD and DS showed lower self-esteem and had lower family QOL, while parents of children with DMT1 did not differ significantly from the CG. It is possible that lower self-esteem in parents of children with disabilities is triggered by higher perceived stress due to deviations in the child’s behavior. Other studies also confirmed the presence of lower self-esteem and life satisfaction in parents of children with ASD [46].

Slightly higher self-esteem was recorded in parents of children with DMT1 and it may be related to the education that parents must undergo in order to acquire specific knowledge and skills related to childcare. Informal education can increase the level of self-esteem, a sense of self-actualization, and parental self-efficacy, which can make a significant contribution to disease control in a child [49,50]. Despite the preserved self-esteem, parents of children with DMT1 were the least optimistic when compared to other groups, which may indicate their constant concern about the future and their fear for the child’s life. It is possible that their general expectation that good things will happen as well as the belief that the future will be favorable are disturbed by constant caution and concern about the threat of hypoglycemia and the fear that their child will have a poor quality of life. Fear of hypoglycemia is in itself associated with increased psychological stress and a poorer quality of life [37,51].

Perceived stress and depressive symptomatology had a significant effect on the individual QOL and the family QOL. It is possible that such conditions, in addition to chronic psychological stress, contribute to an unhealthy lifestyle, further undermining their health and coping mechanisms. Furthermore, this may indicate that the perceived stress and the depressive mood of an individual family member can significantly disrupt family relationships, and negatively affect the perception of parenthood and the overall family QOL. Other studies also confirmed the impaired QOL in parents of children with developmental disabilities, which was in correlation with depressive moods [52]. Our previous study also confirmed that stress is a strong predictor of depression and anxiety [24].

This study adds further knowledge about the association between the individual and the family QOL in parents of children with chronic conditions. We found a substantial association between these two facets of QOL. The association between all of the family QOL domains and the social domain of the individual QOL indicates a significant interrelationship of the way the family functions and the individual relationships within the family, as well as the support of friends. The association between the environment domain of the individual QOL and the physical and material well-being domains of the family QOL points to a contribution of the environment on supporting and securing the well-being, as well as improving the family QOL of families with children with developmental disabilities and DMT1.

Despite our novel and interesting findings, this study has certain limitations that could affect the generalization of the results. This is a cross-sectional study that cannot prove a causal relationship. Furthermore, the sample was convenient and relatively small, which may have affected the study power, especially in the CP group. Parents of children with DS, ASD and DMT1 were commonly involved in associations offering support for parents of children with specific health conditions, while parents of children with CP did not form such an association in the investigated geographical area. Additionally, the diversity of parental groups included in this study is certainly an advantage, but it can also complicate the interpretation of the results.

Despite these limitations, this study provides useful information for both the clinical practice and for further studies. We included a plethora of characteristics of examinees, and both individual and family QOL into the elaborate analysis. Health care professionals, especially nurses, have a key role in numerous activities aiming at mitigating the negative impact of parenting a child with a chronic health condition. Such support would contribute significantly to relieving parental stress and to increasing their QOL. The application of clinical skills, receiving support during treatment, as well as education related to the child’s illness, ubiquity, understanding, empathy and communication are the key components in providing support to the parents. Additionally, parents of these children would highly benefit from strengthening their self-efficacy, self-confidence and self-esteem, as well as from occasional relaxation and respite care, in order to take a break [4].

## 5. Conclusions

This study confirms that parents of children with chronic conditions are more likely to have lower individual and family quality of life, which were correlated. This could have negative repercussions on parental health. Interventions by healthcare professionals should be implemented and aimed not only towards the child, but also towards the parents and the entire family. It is crucial to decrease the challenges of parenting a child with a chronic condition. It is also important to strengthen parental self-esteem because it was found to be strongly associated with the individual perception of the QOL. The impact of child’s disease on families and parental health is often underestimated or unrecognized, while it could provide an important insight into the effect of treatment. Health care institutions, nursing community services and other support institutions should pay greater attention to the parents of children with chronic conditions in order to preserve the health and improve the quality of life of these vulnerable members of the population.

## Figures and Tables

**Table 1:** Quality of life and psychological characteristics according to study group.

	CG (*N* = 82)	DS (*N* = 36)	ASD(*N* = 36)	CP(*N* = 20)	DMT1 (*N* = 43)	Overall *p*	Post Hoc Test *p*-Values ^≠^
Parent’s age (years), M (SD)	38.4 (5.8)	41.3 (6.6)	38.5 (3.7)	41.0 (7.1)	40.8 (5.1)	0.025 †	0.013^CG/DS^ 0.025^CG/DMT1^ 0.037^DS/ASD^
Parent’s gender, N (%)							
father	37 (45.1)	15 (41.7)	10 (27.8)	8 (40.0)	17 (38.6)	0.528 *	/
mother	45 (54.9)	21 (58.3)	26 (73.2)	12 (60.0)	26 (61.4)		
Self-Esteem, M (SD)	23.3 (4.1)	20.6 (4.2)	20.9 (4.0)	21.5 (3.5)	22.8 (5.0)	0.005 †	0.002^CG/DS^ 0.006^CG/ASD^
Optimism, M (SD)	16.4 (3.1)	14.5 (4.3)	15.3 (4.2)	15.3 (3.6)	14.5 (3.9)	0.038 †	0.012^CG/DS^ 0.008^CG/DMT1^
Anxiety, Me (IQR)	4 (5)	3.5 (5)	5.5 (6)	4 (6)	4 (3)	0.539 *	/
Depression, M (SD)	3 (4)	4 (6)	5 (6)	5 (7)	4 (3)	0.042 *	0.011^CG/ASD^ 0.031^CG/CP^ 0.033^CG/DMT1^
Perceived Stress, M (SD)	14.6 (5.3)	15.0 (6.0)	18.1 (5.3)	16.8 (6.4)	16.1 (5.2)	0.021 †	0.002^CG/ASD^
Family Quality of Life, Me (IOR)							
Family Interaction	26.0 (5.0)	24.0 (6.0)	23.0 (5.0)	26.5 (5.0)	27.0 (5.0)	<0.001 *	0.001^CG/DS^ < 0.001^CG/ASD^
Parenting	25.0 (4.0)	23.0 (5.0)	23.0 (3.0)	25.0 (4.0)	24.0 (5.0)	<0.001 *	<0.001^CG/DS^ < 0.001^CG/ASD^
Emotional Well-being	16.0 (4.0)	15.0 (3.0)	14.0 (5.0)	14.5 (3.0)	16.0 (4.0)	<0.001 *	0.015^CG/DS^ < 0.001^CG/ASD^
Physical/Material Well-being	22.0 (4.0)	20.0 (4.0)	19.0 (2.0)	19.5 (6.0)	22.0 (3.0)	<0.001 *	0.003^CG/DS^ < 0.001^CG/ASD^
Disability-Related Support	18.0 (4.0)	16.0 (3.0)	16.0 (5.0)	16.0 (3.0)	18.0 (4.0)	<0.001 *	0.007^CG/DS^ < 0.001^CG/ASD^
Overall FQOL	105.0 (18.0)	100.0 (17.0)	92.5 (16.0)	101.0 (17.0)	109.0 (17.0)	<0.001 *	0.001^CG/DS^ < 0.001^CG/ASD^ 0.102^CG/CP^
Individual Quality of Life, Me (IOR)							
Physical health	16.6 (2.9)	16.3 (2.9)	15.4 (2.9)	15.1 (2.9)	16.6 (2.9)	0.011 *	0.007^CG/ASD^ 0.003^CG/CP^
Psychological well-being	16.0 (2.8)	16.0 (2.5)	14.7 (3.8)	16.0 (3.7)	16.0 (2.0)	0.019 *	0.001^CG/ASD^
Social relationship	16.0 (4.0)	15.3 (2.3)	14.7 (3.7)	16.0 (2.3)	14.7 (2.7)	0.001 *	0.002^CG/DS^ < 0.001^CG/ASD^ 0.029^CG/DMT1^
Environmental support	15.0 (2.5)	14.7 (3.0)	13.5 (3.9)	12.6 (2.9)	15.0 (3.3)	<0.001 *	0.009^CG/DS^ < 0.001^CG/ASD^ < 0.001^CG/CP^
Individual perception QOL and own health	16.0 (2.0)	16.0 (2.0)	14.0 (3.5)	16.0 (2.0)	16.0 (2.0)	0.001 *	0.035^CG/DS^ < 0.001^CG/ASD^ 0.038^CG/CP^

Note: CG—parents of children with typical development and without chronic diseases; DS—parents of children with Down syndrome; ASD—parents of children with autistic spectrum disorder; CP—parents of children with cerebral palsy; DMT1—parents of children with diabetes mellitus type 1; IQR—interquartile range; SD—standard deviation; * Kruskal–Wallis test; † ANOVA; ^≠^ only significant values are shown.

**Table 2:** Association between family quality of life and parents’ characteristics using multivariate linear regression.

	Family Interaction		Parenting		Emotional Well-Being		Physical/Material Well-Being		Disability-Related Support		Overall	
	ß	*p*	ß	*p*	ß	*p*	ß	*p*	ß	*p*	ß	*p*
DS	−0.23	0.008	−0.27	0.005	−0.16	0.082	−0.23	0.010	−0.22	0.026	−0.27	0.001
ASD	−0.21	0.008	−0.32	<0.001	−0.32	<0.001	−0.30	<0.001	−0.44	<0.001	−0.38	<0.001
CP	0.02	0.831	−0.04	0.613	−0.04	0.583	−0.08	0.247	0.04	0.656	−0.04	0.546
DMT1	−0.15	0.120	−0.22	0.032	−0.11	0.291	−0.08	0.386	−0.18	0.085	−0.18	0.041
Fathers	0.02	0.850	−0.11	0.196	−0.08	0.335	0.01	0.943	−0.03	0.769	−0.06	0.463
Age	−0.05	0.488	−0.02	0.844	−0.12	0.140	−0.04	0.561	−0.05	0.669	−0.06	0.344
High school	−0.10	0.231	−0.10	0.235	−0.14	0.118	−0.18	0.025	−0.25	0.007	−0.18	0.018
Monthly income <1400 EUR	−0.20	0.015	−0.18	0.042	−0.07	0.416	−0.01	0.893	0.00	0.983	−0.12	0.115
Association’s member (no)	0.15	0.094	0.14	0.163	0.16	0.113	0.20	0.027	0.14	0.153	0.19	0.027
No active smoking	0.01	0.189	−0.13	0.081	0.00	0.987	−0.02	0.790	−0.05	0.511	−0.05	0.486
Chronic diseases (no)	−0.03	0.665	−0.06	0.400	−0.04	0.581	−0.03	0.669	−0.02	0.820	−0.04	0.500
Body Mass Index	0.05	0.670	0.06	0.731	−0.03	0.746	−0.05	0.511	0.09	0.323	0.03	0.639
Activity	−0.03	0.647	−0.04	0.638	0.01	0.864	−0.05	0.519	0.02	0.784	−0.02	0.757
Mediterranean Diet	0.17	0.012	0.05	0.475	0.03	0.648	0.00	0.951	0.00	0.993	0.07	0.275
Caring for children (h/d)	0.21	0.004	0.21	0.006	0.20	0.010	0.20	0.005	0.11	0.144	0.23	0.001
Optimism	0.11	0.149	0.09	0.271	−0.06	0.450	0.09	0.236	0.03	0.714	0.07	0.335
Self-Esteem	0.38	<0.001	0.23	0.013	0.16	0.090	0.13	0.141	0.04	0.648	0.24	0.003
Anxiety	0.38	0.002	0.25	0.049	0.30	0.023	0.32	0.008	0.27	0.042	0.37	0.001
Depression	−0.08	0.493	−0.26	0.045	−0.25	0.059	−0.31	0.012	−0.10	0.457	−0.23	0.042
Perceived Stress	−0.36	0.001	−0.15	0.183	−0.39	0.001	−0.38	0.001	−0.41	0.001	−0.41	<0.001

Note: ß—Standardized beta; *p*—*p*-value; parents in control group are reference group; DS—parents of children with Down syndrome; ASD—parents of children with autistic spectrum disorder; CP—parents of children with cerebral palsy; DMT1—parents of children with diabetes mellitus type 1; h/d—hour per day.

**Table 3:** Association between individual quality of life and parents’ characteristics using multivariate linear regression.

	Psychical Health		Psychological Well-Being		Social Relationships		Environment		Individual Perception QOL and Own Health	
	ß	*p*	ß	*p*	ß	*p*	ß	*p*	ß	*p*
DS	−0.02	0.766	0.01	0.840	0.01	0.593	−0.03	0.685	−0.02	0.772
ASD	0.00	0.985	−0.10	0.096	−0.19	0.026	−0.12	0.086	−0.16	0.024
CP	−0.04	0.536	0.01	0.862	0.01	0.917	−0.16	0.010	−0.13	0.040
DMT1	−0.03	0.693	−0.04	0.567	0.03	0.797	0.12	0.140	−0.12	0.171
Parent’s age	0.03	0.473	0.01	0.900	−0.07	0.370	−0.05	0.411	−0.13	0.051
Fathers	−0.10	0.190	−0.07	0.253	−0.05	0.567	−0.08	0.271	−0.14	0.061
High school	−0.02	0.773	−0.05	0.454	−0.01	0.944	−0.11	0.123	−0.04	0.573
Monthly income <1400 EUR	0.00	0.966	−0.04	0.559	0.03	0.686	0.19	0.009	0.02	0.806
Association’s member (no)	−0.06	0.451	−0.01	0.929	−0.07	0.432	−0.08	0.326	0.07	0.400
No active smoking	0.00	0.949	−0.07	0.224	−0.06	0.448	−0.01	0.847	0.08	0.190
Chronic diseases (no)	0.23	<0.001	−0.03	0.513	−0.07	0.330	0.03	0.622	0.13	0.031
Body Mass Index	−0.01	0.848	−0.10	0.091	0.01	0.912	−0.04	0.531	−0.16	0.027
Activity	−0.09	0.717	−0.02	0.757	0.01	0.847	0.02	0.687	0.01	0.936
Mediterranean Diet	0.11	0.081	0.06	0.246	0.06	0.419	0.16	0.007	0.01	0.870
Caring for children (h/d)	0.06	0.393	0.06	0.256	0.18	0.018	0.01	0.932	0.09	0.141
Optimism	−0.09	0.213	0.06	0.321	0.05	0.532	0.05	0.502	0.25	0.001
Self-Esteem	0.27	0.001	0.59	<0.001	0.42	<0.001	0.26	0.001	0.17	0.027
Anxiety	−0.01	0.961	−0.10	0.299	0.08	0.520	0.06	0.592	−0.21	0.053
Depression	−0.22	0.053	0.05	0.626	0.04	0.766	−0.10	0.346	0.05	0.643
Perceived Stress	−0.26	0.012	−0.22	0.008	−0.19	0.102	−0.35	<0.001	−0.23	0.018

Note: ß—Standardized beta; *p*—*p*-value; parents in control group are reference group; DS—parents of children with Down syndrome; ASD—parents of children with autistic spectrum disorder; CP—parents of children with cerebral palsy; DMT1—parents of children with diabetes mellitus type 1; h/d—hour per day.

## Data Availability

The data that support the findings of this study are available from the corresponding author, upon reasonable request.

## Supplementary Materials

The following supporting information can be downloaded at: https://www.mdpi.com/article/10.3390/jcm11102861/s1, Table S1: Family composition, sociodemographic, health and lifestyle characteristics of parents; Table S2: Relationship between family and individual quality of life in the overall sample including all parents (using Pearson correlation test).
