# Supplementary material for: Family and Individual Quality of Life in Parents of Children with Developmental Disorders and Diabetes Type 1
Source: J Clin Med. 2022 May 19;11(10):2861. doi: 10.3390/jcm11102861 (PMC9145317; doi:10.3390/jcm11102861)
Supplement: Supplementary file 1 [file jcm-11-02861-s001.zip › jcm-1714216-SI.pdf]

Supplemental Table S1. Family composition, sociodemographic, health and lifestyle characteristics of parents

	CG (N=82)	DS (N=36)	ASD (N=36)	CP (N=20)	DMT1 (N=43)	Overall p
Parent's education, N (%)						
high school	25 (30.5)	27 (75.0)	20 (54.3)	14 (70.0)	30 (69.8)	
bachelor degree	16 (19.5)	5 (13.9)	5 (14.3)	2 (10.0)	7 (16.3)	<0.001*
master degree	41 (50.0)	5 (11.1)	11 (31.4)	4 (20.0)	6 (14.0)	
Working status, N (%)						
employed full time	72 (87.8)	20 (55.6)	16 (44.4)	11 (55.0)	32 (72.6)	
disability benefit status	0 (0.0)	6 (16.7)	11 (30.6)	6 (30.0)	5 (11.9)	<0.001*
unemployed	10 (12.2)	10 (27.8)	9 (25.0)	3 (15.0)	5 (11.9)	
Monthly income, N (%)						
<800 €	3 (3.8)	4 (11.1)	8 (22.2)	2 (10.0)	10 (23.8)	
800 –1.400 €	15 (19.0)	17 (47.2)	9 (25.0)	13 (65.0)	12 (28.6)	<0.001*
1.400 – 2.000 €	28 (41.8)	11 (30.6)	14 (38.9)	4 (20.0)	14 (33.3)	
>2.000 €	28 (35.4)	4 (11.1)	5 (13.9)	1 (5.0)	6 (14.3)	
Number of children, N (%)						
one	13 (15.9)	4 (11.1)	8 (22.2)	8 (40.0)	6 (14.0)	
two	56 (68.3)	15 (41.7)	20 (55.6)	5 (25.07)	28 (65.1)	0.001*
three or more	13 (15.9)	17 (47.2)	8 (22.2)	7 (35.0)	9 (20.9)	
Child's age (years), M (SD)	6.4 (2.0)	7.3 (2.4)	7.5 (2.0)	8.6 (2.8)	9.5 (1.8)	<0.001†
Child's gender, N (%)						
boys with chronic conditions	-	14 (38.9)	33 (91.7)	11 (55.0)	29 (67.4)	<0.001*
girls with chronic conditions	-	22 (61.1)	3 (8.3)	9 (45.0)	14 (32.6)	
typical development boys	98 (58.3)	35 (66.0)	21 (60.0)	11 (44.0)	23 (51.1)	0.363*
typical development girls	70 (41.7)	18 (34.0)	14 (40.0)	14 (56.0)	22 (48.9)	
Caring for children; (hours/day), Me (IQR)						
care for child with chronic conditions	-	3.0 (2.0)	4.5 (3.0)	5.0 (2.0)	4.0 (3.0)	0.085‡
care for typical development children	4.0 (2.0)	3.0 (1.0)	3.0 (1.0)	3.0 (4.0)	3.0 (2.0)	<0.001‡
Close friends, Me (IQR)	4.0 (3.0)	3.0 (3.0)	2.0 3.0)	3.0 (5.0)	4.0 (3.0)	0.002‡
Association's members, N (%)	10 (8.0)	26 (74.3)	15 (42.9)	2 (10.5)	32 (82.1)	<0.001*
Body Mass Index, M (SD)	24.6 (3.3)	25.4 (4.7)	24.9 (4.3)	25.7 (4.4)	25.2 (4.4)	0.807*
Chronic illness, N (%)	37 (45.1)	20 (55.6)	18 (50.0)	13 (65.0)	21 (48.8)	0.551*
Health self-assessment, N (%)						
bad	1 (1.3)	1 (3.1)	2 (5.7)	3 (15.0)	1 (2.4)	
good	67 (83.8)	24 (75.0)	28 (80.0)	14 (70.0)	37 (90.2)	0.148*
very good	12 (15.0)	7 (21.9)	5 (14.3)	3 (15.0)	3 (7.3)	
Mediteranean diet, M (SD)	7.9 (4.3)	8.2 (3.8)	8.3 (3.6)	7.3 (4.1)	8.4 (4.3)	0.884†
Sitting (hours/day), Me (IQR)	5.0 (4.3)	3.0 (3.3)	4.0 (3.8)	4.5 (5.8)	4.0 (2.8)	0.186‡
Alcohol consumption, N (%)	35 (42.7)	12 (33.3)	9 (25.0)	7 (35.0)	13 (30.2)	0.382*
Smoking, N (%)	25 (30.5)	11 (31.4)	17 (47.2)	4 (20.0)	17 (39.5)	0.226*

Note: CG-parents of children with typical development and without chronic diseases; DS-parents of children with Down syndrome; ASD-parents of children with autistic spectrum disorder; CP-parents of children with cerebral palsy; DMT-parents of children with diabetes mellitus type 1; M-mean; SD-standard deviation; Me-median; IQR-interquartile range; *Chi-square test; †ANOVA; ‡Kruskal-Wallis test.

Supplemental Table S2. Relationship between family and individual quality of life in the overall sample including all parents (using Pearson correlation test) *

	**FQOL-D1**	**FQOL_D2**	**FQOL_D3**	**FQOL_D4**	**FQOL_D5**	**FQOL_D6**	**WHO_D1**	**WHO_D2**	**WHO_D3**	**WHO_D4**	**WHO_D5**
FQoL_D1	1.000	0.813	0.621	0.695	0.561	0.866	0.345	0.578	0.559	0.443	0.407
		<0.001	<0.001	<0.001	<0.001	<0.001	<0.001	<0.001	<0.001	<0.001	<0.001
FQoL_D2		1.000	0.680	0.740	0.581	0.894	0.367	0.520	0.558	0.436	0.423
			<0.001	<0.001	<0.001	<0.001	<0.001	<0.001	<0.001	<0.001	<0.001
FQoL_D3			1.000	0.598	0.543	0.798	0.434	0.533	0.500	0.412	0.470
				<0.001	<0.001	<0.001	<0.001	<0.001	<0.001	<0.001	<0.001
FQoL_D4				1.000	0.600	0.845	0.481	0.533	0.539	0.609	0.519
					<0.001	<0.001	<0.001	<0.001	<0.001	<0.001	<0.001
FQoL_D5					1.000	0.777	0.330	0.425	0.420	0.398	0.391
						<0.001	<0.001	<0.001	<0.001	<0.001	<0.001
FQoL_D6						1.000	0.458	0.600	0.580	0.531	0.509
							<0.001	<0.001	<0.001	<0.001	<0.001
WHO_D1							1.000	0.517	0.419	0.561	0.577
								<0.001	<0.001	<0.001	<0.001
WHO_D2								1.000	0.643	0.605	0.635
									<0.001	<0.001	<0.001
WHO_D3									1.000	0.570	0.540
										<0.001	<0.001
WHO_D4										1.000	0.623
											<0.001
WHO_D5											1.000

Note: FQOL_D1=family interaction domain; FQOL_D2=parenting domain; FQOL_D3=emotional well-being domain; FQOL_D4=physical/material well-being domain; FQOL_D5=disability-related support domain; FQOL_D6=Overall FQOL (sum of scores for all domains); WHO_D1=psychical health domain; WHO_D2=psychological domain; WHO_D3=Social relationship domain; WHO_D4=environment domain; WHO_D5=overall individual perception QoL and own health; *Recoded Spearman's rho correlation coefficients and p values (non-parametric partial correlation was used to adjust for gender and age).
